# Exercise Decreases Lipogenic Gene Expression in Adipose Tissue and Alters Adipocyte Cellularity during Weight Regain After Weight Loss

**DOI:** 10.3389/fphys.2016.00032

**Published:** 2016-02-10

**Authors:** Erin D. Giles, Amy J. Steig, Matthew R. Jackman, Janine A. Higgins, Ginger C. Johnson, Rachel C. Lindstrom, Paul S. MacLean

**Affiliations:** ^1^Anschutz Health and Wellness Center, University of Colorado Anschutz Medical CampusAurora, CO, USA; ^2^Division of Endocrinology, Diabetes and Metabolism, Department of Medicine, University of Colorado Anschutz Medical CampusAurora, CO, USA; ^3^Department of Pediatrics, University of Colorado Anschutz Medical CampusAurora, CO, USA

**Keywords:** energy balance, obesity, lipid trafficking, *de novo* lipogenesis, adipogenesis

## Abstract

Exercise is a potent strategy to facilitate long-term weight maintenance. In addition to increasing energy expenditure and reducing appetite, exercise also favors the oxidation of dietary fat, which likely helps prevent weight re-gain. It is unclear whether this exercise-induced metabolic shift is due to changes in energy balance, or whether exercise imparts additional adaptations in the periphery that limit the storage and favor the oxidation of dietary fat. To answer this question, adipose tissue lipid metabolism and related gene expression were studied in obese rats following weight loss and during the first day of relapse to obesity. Mature, obese rats were weight-reduced for 2 weeks with or without daily treadmill exercise (EX). Rats were weight maintained for 6 weeks, followed by relapse on: (a) *ad libitum* low fat diet (LFD), (b) *ad libitum* LFD plus EX, or (c) a provision of LFD to match the positive energy imbalance of exercised, relapsing animals. 24 h retention of dietary- and *de novo*-derived fat were assessed directly using ^14^C palmitate/oleate and ^3^H_2_0, respectively. Exercise decreased the size, but increased the number of adipocytes in both retroperitoneal (RP) and subcutaneous (SC) adipose depots, and prevented the relapse-induced increase in adipocyte size. Further, exercise decreased the expression of genes involved in lipid uptake (CD36 and LPL), *de novo* lipogenesis (FAS, ACC1), and triacylglycerol synthesis (MGAT and DGAT) in RP adipose during relapse following weight loss. This was consistent with the metabolic data, whereby exercise reduced retention of *de novo*-derived fat even when controlling for the positive energy imbalance. The decreased trafficking of dietary fat to adipose tissue with exercise was explained by reduced energy intake which attenuated energy imbalance during refeeding. Despite having decreased expression of lipogenic genes, the net retention of *de novo*-derived lipid was higher in both the RP and SC adipose of exercising animals compared to their energy gap-matched controls. Our interpretation of this data is that much of this lipid is being made by the liver and subsequently trafficked to adipose tissue storage. Together, these concerted effects may explain the beneficial effects of exercise on preventing weight regain following weight loss.

## Introduction

There is no doubt that obesity has reached epidemic rates worldwide, and obesity increases the risk of many diseases, including type 2 diabetes, cardio-metabolic disease, as well as cancer. Although losing weight has been shown to improve obesity-associated comorbidities, maintaining a lower body weight is difficult for many individuals, with less than 20% of overweight or obese individuals able to maintain a 10% reduction in body weight 1 year after weight loss (Wing and Phelan, 2005; Kraschnewski et al., 2010).

A significant barrier to weight loss maintenance is the concerted adaptive response that results in an increased appetite and a suppression of energy expenditure (Rosenbaum et al., 2010; Maclean et al., 2011). In animal models, this biological drive to regain weight has been shown to be extremely strong and persistent (Levin and Keesey, 1998; Levin and Dunn-Meynell, 2000; MacLean et al., 2004a,b), and may underlie the high recidivism rates observed with obesity. It is very likely that any strategies that hope to facilitate success with long-term weight maintenance will need to acknowledge this powerful biological response and incorporate ways to ameliorate it.

There is growing evidence that exercise has benefits for facilitating weight loss maintenance (Schoeller et al., 1997; Weinsier et al., 2002; Catenacci et al., 2011). However, it is becoming clear that weight loss and weight loss maintenance represent two very distinct metabolic states (MacLean et al., 2015a), and there are good reasons to explain why the effects of regular exercise may be different for the two objectives. In our rodent models of weight regain, regular exercise increases energy expenditure and decreases appetite (Steig et al., 2011), and these effects have been linked to an increased capacity to oxidize dietary fat and the trafficking of ingested carbohydrate through a more energetically expensive pathway of deposition: *de novo* lipogenesis. Further, in the context of weight loss maintenance, we have shown that regular exercise enhances the expression of genes involved in mitochondrial biogenesis, beta-oxidation, and the uptake and utilization of lipid in skeletal muscle (Steig et al., 2011).

Adipose tissue is known to play an important role in the biological drive to regain weight (Dulloo and Montani, 2015). Thus, in the present investigation, we examined the impact of regular exercise on the metabolic state of adipose tissues during weight loss maintenance and during weight regain. In both the parent study and this analysis, we included a “gap-matched” control group, in which weight reduced sedentary rats were allowed to overfeed to the same extent as weight reduced rats that were exercised on a daily basis. This novel study design allowed us to observe any adaptive response in adipose tissue that extended beyond the ability of exercise to reduce the positive energy imbalance during weight regain. We hypothesized that regular exercise would reduce the lipogenic capacity of adipose tissue, facilitating the trafficking of ingested fat toward oxidative pathways in muscle and away from pathways of lipid deposition in adipose tissue. Such observations would suggest that the beneficial effects of exercise on whole body metabolism and energy balance during weight regain are the result of concerted effects on peripheral tissues.

## Materials and methods

### Experimental paradigm of weight regain

One hundred and twenty Male Wistar rats (125–150 g; 5 weeks of age) were purchased from Charles River Laboratories (Charles River Laboratories, Inc., Wilmington, MA) and were housed in the University of Colorado Anschutz Medical Campus Center for Comparative Medicine and the Center for Human Nutrition Satellite Animal Facility (22–24°C; 12:12 h light-dark cycle) with free access to water. All procedures were approved by the institutional animal care and use committee. Rats were individually housed in wire bottom metabolic cages that limit activity (relative to group housed animals in polycarbonate cages), and obesity prone rats (*n* = 56) were identified by their propensity to gain weight following *ad libitum* access to a high-fat diet (HFD, 46% kcal fat 21% kcal protein, 33% kcal carbohydrate Research Diets, Inc., New Brunswick, NJ; RDI# D12344) from 5 to 6 weeks of age. Rats whose weight increased by ≥30% over 1 week were classified as obesity prone. We have previously shown that this screening process is predictive of the rat's tendency for future weight gain (Hill, 1990).

**Figure 1:**
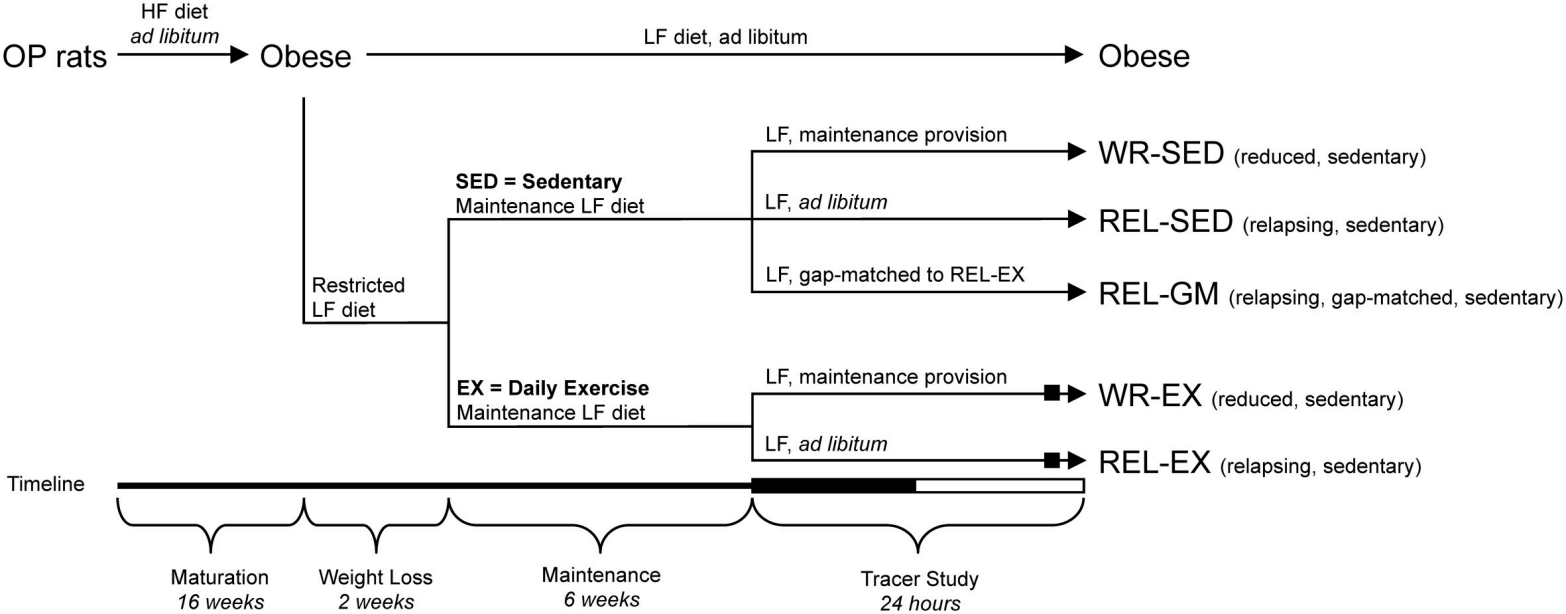
**Experimental groups and tracer study design**. Study Design to generate the groups to investigate effect of exercise [sedentary (SED) vs. exercise (EX)] in weight-reduced (WR) and relapsing (REL) conditions. The REL-GM (relapsing, gap-matched) group was a sedentary group of weight-reduced rats that were given a specific amount of food on the tracer day, so that their positive energy imbalance matched that of the REL-EX group. Rats were placed in the metabolic monitoring system for the final 4 days of the study. The 12:12-h dark-light cycle of the final tracer day is indicated on the timeline (black bar and white bar, respectively). The WR-EX and REL-EX groups performed their regular bout of exercise on the final tracer day (black box), within 3 h of the end of their final light cycle.

An overview of the study design is depicted in Figure 1. To induce obesity (~30–35% body fat), obesity-prone rats were matured for 12 additional weeks in this obesogenic environment (HFD plus limited physical activity). Once animals had developed obesity, a cohort of obese rats (*n* = 8) was switched to *ad libitum* access to a low fat diet (LFD, 25% kcal fat, 21% kcal protein, 54% kcal carbohydrate; Research Diets, Inc., New Brunswick, NJ; RDI# D07091301) for the duration of the study (Obese group; OB). The remaining 48 rats underwent weight reduction via restricted provisions of LFD equivalent to ~60% of the calories they had eaten *ad libitum*. This weight loss regimen was maintained for 2 weeks, causing a 14–18% loss in total body mass. Animals were maintained at this reduced weight for 6 weeks by providing a limited provision of LFD at the beginning of each dark cycle (14:00). Animals were weighed twice per week and energy intakes were adjusted accordingly to maintain weight stability. During the weight reduction and stability phases, 16 of the 48 rats performed regular exercise bouts.

### Experimental groups

Prior to the weight loss phase, animals were stratified according to body weight to ensure a similar weight distribution into each of the six experimental groups (Figure 1). These groups included: obese (OB), weight-reduced sedentary (WR-SED), weight-reduced exercise (WR-EX), relapsing sedentary (REL-SED), relapsing exercise (REL-EX), and energy gap-matched to REL-EX (REL-GM). On the final testing day, the WR-SED (*n* = 9) and WR-EX (*n* = 7) control rats were given their daily bolus of LFD to maintain energy balance as described above. REL-SED (*n* = 8) and REL-EX (*n* = 7) rats were allowed *ad libitum* access to the LF diet throughout the final 24-h testing period in order to examine the initial day of relapse to obesity. Both WR-EX and REL-EX animals participated in their regular bout of physical activity on the testing day. To determine if differences between sedentary (SED) and exercising (EX) rats were due to differences in the energy imbalance or to independent effects on peripheral tissues, we included an additional group of sedentary weight-reduced rats that were allowed to overfeed only to the point at which their energy imbalance was the same as REL-EX rats (matching their energy gap; gap-matched, REL-GM). Obese rats (OB; *n* = 8) had *ad libitum* access to the LFD during the entire 24-h testing period. All animals were sacrificed at the end of the 24 h testing day.

### Exercise regimen

Sixteen rats (WR-EX and REL-EX) performed regular exercise bouts throughout the 8 weeks of weight reduction and maintenance. Rats exercised on a three-lane Exer-6M Treadmill (Columbus Instruments, Columbus, OH) during the 8 weeks of weight loss and maintenance. Rats were acclimated to the treadmill by incrementally increasing the exercise intensity from 5 to 15 m/min during the first week of training and then increasing the exercise duration from 10 to 60 min during the second week. For the remainder of the study, rats performed regular exercise bouts for 1 h a day, 6 days a week, at an intensity of 15 meters/min. Throughout the study, the exercise bout was performed at approximately the same time of day (between 11:00 a.m. and 2:00 p.m., in the 3 h window prior to the onset of the dark cycle).

Rats were encouraged to perform the daily exercise bouts using one or more of the following methods: (1) placing food pellets or dangling a novel play item at the head of the treadmill so it was just out of reach of the animal; (2) a short shock from an electric grid at the rear of the treadmill (10V, 0.5A, 0.75hz); (3) applying a bristle brush to the animal's feet on the rear treadmill grid; or (4) intermittent air puffs to the hind-quarters. The type and combination of motivation used varied depending on the rat's response to the different motivation techniques.

### Energy balance and fuel utilization

A metabolic monitoring system (Columbus Instruments, Columbus, OH) was used to assess energy balance and fuel utilization throughout the final testing day. This multichamber indirect calorimetry system allows for the continuous monitoring of up to eight rats, obtaining measurements of oxygen consumption (vO_2_) and carbon dioxide production (vCO_2_) from each chamber every 16 min (MacLean et al., 2004b; Jackman et al., 2008). The chambers also allow for the collection of daily urine, feces, and food spillage. Energy intake (EI) was measured every 3 h during the 24-h tracer protocol (described below), taking into account any spilled food. Metabolic rate (MR) was calculated from gas exchange measurements using the Weir equation (MR = 3.941 * vO_2_ + 1.106 * vCO_2_ − 2.17 * N), where N represents urinary nitrogen. This was then used to calculate total energy expenditure (TEE), component analysis of TEE and to acquire estimates of non-resting (NREE) and resting (REE) energy expenditure as previously described (MacLean et al., 2004a,b; Jackman et al., 2008; Giles et al., 2010). Respiratory Exchange Ratio (RER) was calculated as the ratio of CO_2_ production to O_2_ consumption (vCO_2_/vO_2_). Energy balance (EB) was calculated as EI – TEE.

### Dual tracer and testing day protocol

The dual-tracer approach was similar to our previous work in sedentary rats (Jackman et al., 2008; Giles et al., 2010; Figure 1). After 2 days of acclimatization in the metabolic chambers, a 250 μCi intraperitoneal injection of ^3^H_2_0 was given 2 h prior to start of the final dark cycle. Previous studies have shown that this tracer equilibrates with total body water within 2 h and remains relatively steady over the next 24-h period (Commerford et al., 2001). Subsequent incorporation of tritium into lipid pools provides an estimate of the net retention of carbons, but does not provide any information as to the carbon source.

To reflect the dietary fat within the LFD, 1-[^14^C]palmitate and 1-[^14^C]oleate tracers were blended into the diet (1:3 ratio, reflecting their ratio in the diet) at a specific activity of 0.8 μCi/kcal of dietary lipid. The radiolabeled diet was fed at the start of the final dark cycle. Although different groups were provided different amounts of food, and thus different amounts of the ^14^C tracer, the specific activity of the diet was constant. Therefore, the absolute amount of tracer consumed reflected the amount of dietary lipid ingested over the 24-h period. Energy intake (EI) was assessed every 3 h, whereas O_2_ consumption and CO_2_ production were monitored every 16 min by indirect calorimetry. To estimate dietary fat oxidation, effluent CO_2_ from each chamber was collected into 3.0 mL aliquots of a 2:1 mixture of methanol and methylbenzethonium (hyamine) hydroxide (Sigma-Aldrich, St. Louis, MO #B2156) to trap 1 mmol CO_2_. CO_2_ trapping was conducted when the rat was not being monitored by the calorimetry system. The ^14^C content was then measured with a Beckman LS6500 scintillation counter. Background activity was determined by counting a sample containing only scintillation fluid and hyamine hydroxide, and was subtracted from experimental values.

At the end of the 24-h period, rats were anesthetized with isoflurane. A sample of blood was obtained from the vena cava, and rats were then exsanguinated. Retroperitoneal (RP) and subcutaneous (SC) adipose tissues (from the dorsal lumbar region) were collected for determination of ^14^C and ^3^H accumulation in the lipid fraction, as previously described (Jackman et al., 2008). Samples of serum and urine were dried, and ^14^C content was determined via scintillation counting. Total serum ^14^C content was calculated as the measured ^14^C activity/mL of serum × 0.0385 × body mass (grams) (Caster et al., 1956).

### Gene expression analyses

Total RNA was extracted from powdered adipose tissue using TRIzol (Invitrogen). RNA concentration was determined using a Nanodrop 1000 Spectrophotometer system (Thermo Scientific, Wilmington, DE; software version 3.2.1), and RNA integrity was verified with an Agilent 2100 Bioanalyzer (Agilent Technologies, Santa Clara, CA). Total RNA (1 μg) was reverse transcribed with the iScript cDNA synthesis kit (Bio-Rad, Hercules, CA), and quantitative PCR was performed using primer sets for genes of interest and reference genes, and iQ SYBR Supermix (Bio-Rad) following manufacturer protocols. Reactions were run in duplicate on an iQ5 real-time PCR detection system (Bio-Rad) along with a no-template control for each gene. Validation experiments were performed to demonstrate that efficiencies of target and reference genes were approximately equal. The target genes were normalized to the geometric means of ubiquitin C using the comparative Ct method (Vandesompele et al., 2002).

### Cellularity

At the time of sacrifice, RP and SC fat pads were removed and weighed, and a portion of each fat pad was immediately processed for cellularity by methods described previously (Jackman et al., 2008; MacLean et al., 2009; Giles et al., 2012). In brief, a portion of the fat pad was suspended in cold Krebs-Ringer phosphate (KRP) buffer and minced. Samples were then digested with collagenase, stained with methylene blue, and adipocytes (150–250 cells/sample) were imaged by a blinded microscopist using an Olympus BX60 microscope and a C-mounted Canon Power Shot G5 digital camera. Images were analyzed with a Cell Counting Analysis Program (Mayo Clinic, Rochester, MN) to obtain the diameter. The number of cells per fat pad was calculated from the average adipocyte volume, a density conversion factor (0.915 g/ml), and the mass of the fat pad.

### Statistical analysis

Data were analyzed using SPSS software version 23.0 by analysis of variance with planned comparisons examining whether exercise alters energy homeostasis in weight-reduced animals (RED-SED vs. RED-EX), during the first day of relapse (REL-SED vs. REL-EX), and during relapse if the energy imbalance is controlled (REL-EX vs. REL-GM). Differences in adipocyte cell frequency distribution were examined by chi-square analysis for the three aforementioned planned comparisons, with a *z*-test (Bonferroni's adjustment) to determine differences in proportions. Statistical significance was set at *p* < 0.05.

## Results

**Figure 2:**
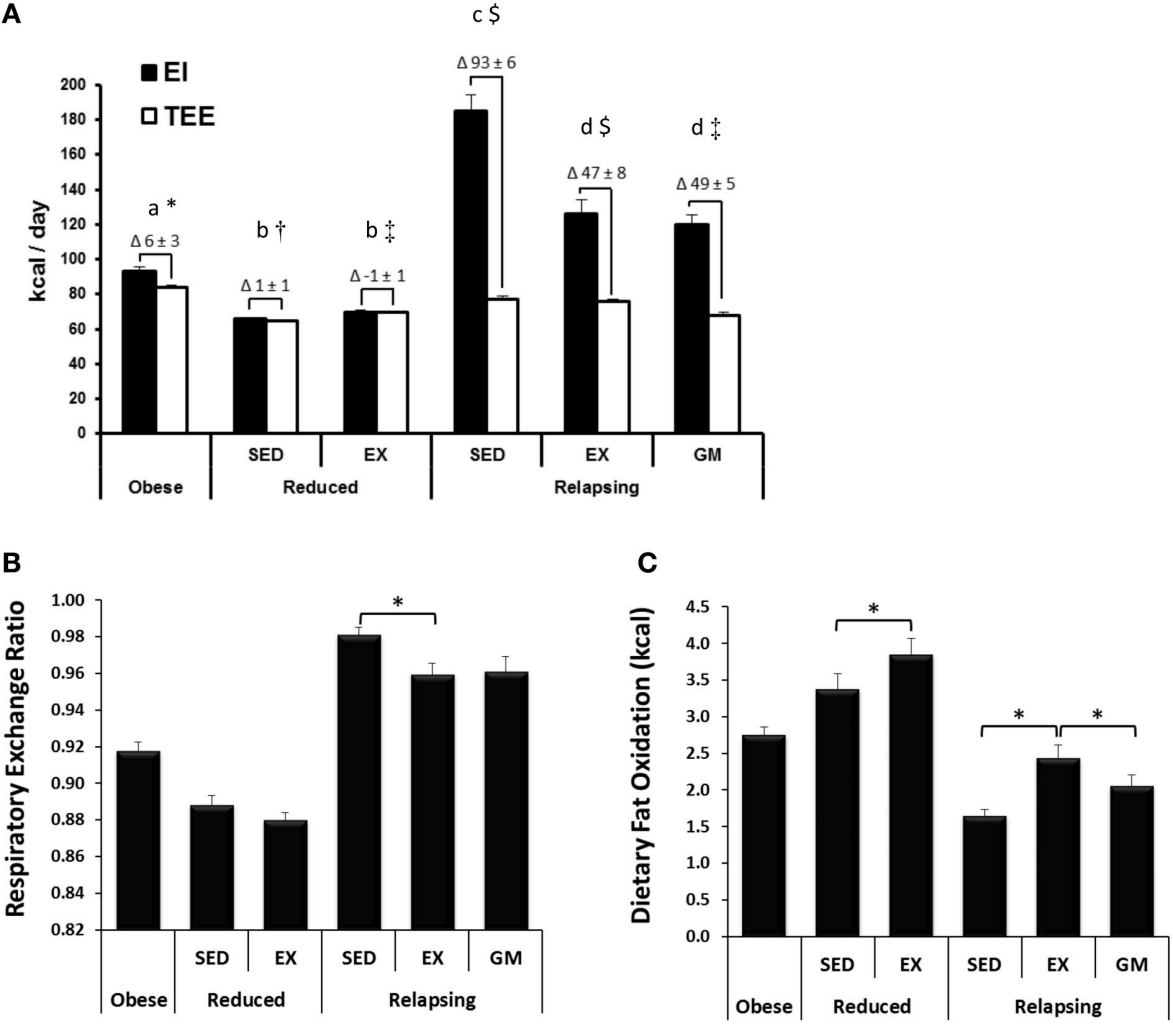
**Energy Balance, Respiratory Exchange Ratio, and Whole Body Dietary Fat Oxidation**. **(A)** Energy intake (EI) and total energy expenditure (TEE), as measured by indirect calorimetry on the final day of the study are shown, with the calculated energy imbalance indicated as the Δ above each graph. Obese and relapsing SED and EX rats ate *ad libitum*. Reduced rats were given an energy balance provision of the diet. Relapsing gap-matched (REL-GM) were given just enough food to match the energy imbalance of the REL-EX group. Groups designated by the same letter (a, b, c, d for EI) or symbol (^*^, †, ‡, $ for TEE) are not significantly different from one another (*P* < 0.05). **(B)** Exercise decreases respiratory exchange ratio (RER) indicating an increase in whole body fat oxidation in relapsing animals. **(C)** Exercise increases 24 h dietary fat oxidation (measured with a ^14^C-oleate/palmitate dietary tracer) in both reduced and relapsing animals. Figures were modified or derived from the parent study (Steig et al., 2011).

**Figure 3:**
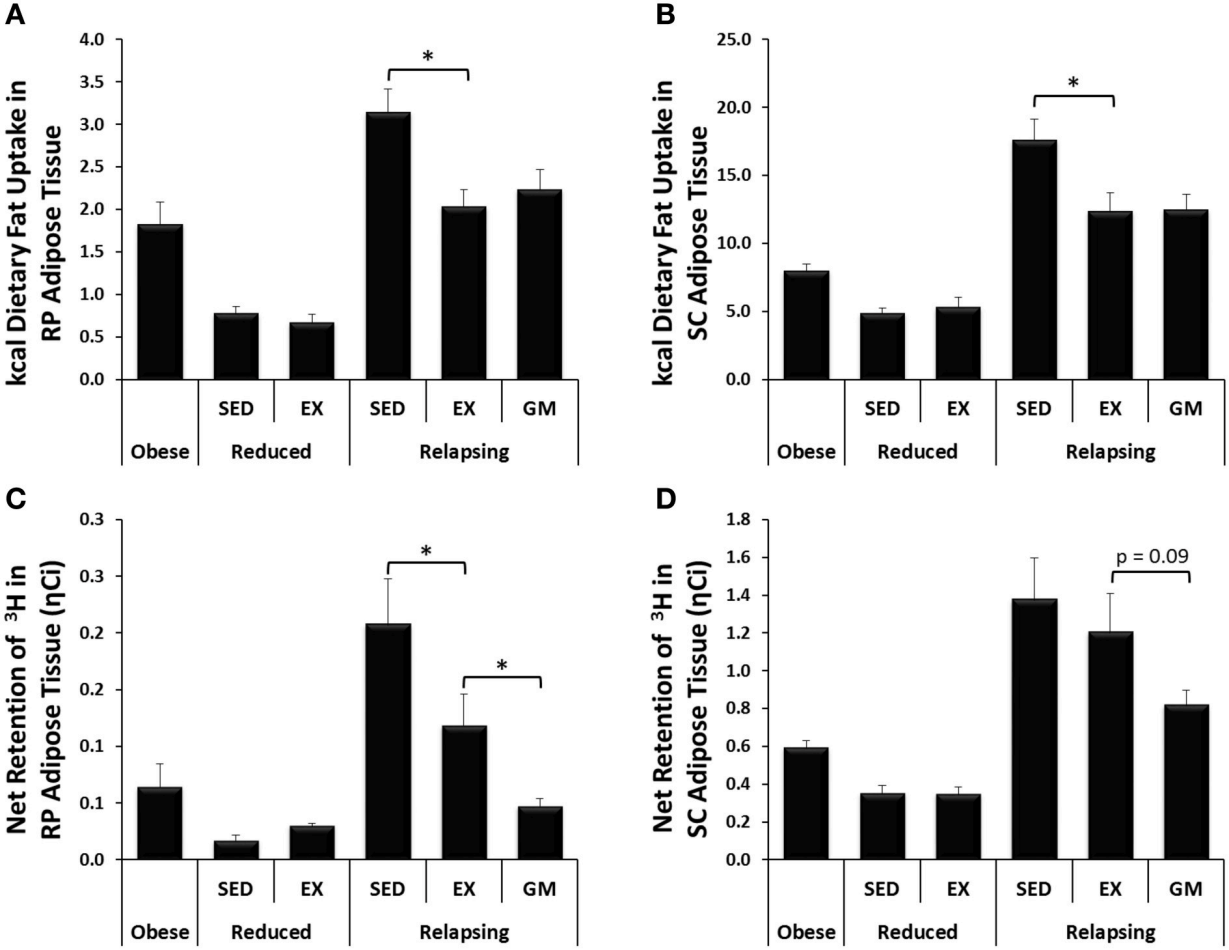
**Trafficking of dietary fat and ***de novo*** lipogenesis**. **(A,B)** In relapsing animals, exercise decreases trafficking of dietary fat (^14^C) to RP and SC adipose depots. **(C,D)** Exercise decreases lipogenesis (^3^H incorporation) in RP and SC adipose during relapse. (^*^P < 0.05).

Body weight, body composition, energy balance, and fat oxidation data from this study has been reported in detail previously (Steig et al., 2011). Key data from the parent study that are required to interpret the results of the current study have been reproduced in Figure 2. In addition, dietary fat retention and *de novo* lipogenesis in RP adipose tissue were presented in the original study on a per gram of adipose tissue. We have extrapolated these data to the entire fat pad and also included identical measurements from SC adipose in the current study (Figure 3) to help build a better picture of the effects of exercise on whole body lipid trafficking and storage.

### Energy balance and gap-matching

The development of obesity, both in terms of body weight and composition, in obesity-prone rats was reflective of that previously published for this model (Jackman et al., 2006, 2008; MacLean et al., 2006). Energy intake (EI) and TEE during the 24 h tracer study are shown in Figure 2A, with the calculated energy imbalance indicated as the Δ above each graph. WR-SED and WR-EX animals were given a provision of the LF diet sufficient to maintain energy balance. Relapsing animals were given *ad libitum* access to the LF diet and, during the relapse period, REL-SED animals consumed ~100 kcal in excess of what they expended. REL-EX experience a 46.9 ± 7.5 kcal energy excess. Because the energy expenditure of the sedentary REL-GM group was ~10 kcal less than their exercising counterparts, they were also provided ~10 kcal less food so that their EB (49.1 ± 5.1 kcal) was not different than the REL-EX groups.

### Whole body and dietary fat oxidation

Despite having no impact on whole body substrate oxidation, reflected in RER (Figure 2B), exercise increased the oxidation of dietary fat in the weight reduced state (Figure 2C; WR-SED vs. WR-EX). During relapse, RER predictably reflected the differences in energy balance in the three relapsing groups, with an increase in RER in the REL-SED animals, who had a greater caloric excess than the REL-EX and REL-GM. However, the effect of exercise on dietary fat oxidation persisted during relapse, with increased dietary fat oxidation in the REL-EX rats, compared to REL-SED. Dietary fat oxidation was significantly lower in the REL-GM compared to REL-EX, indicating that exercise works peripherally to increase oxidation of dietary fat, and the increased oxidation of dietary fat in the exercising animals was not simply the result of exercise-induced changes in energy balance.

### Trafficking of dietary fat to adipose

In addition to providing measurements of dietary fat oxidation, use of radioactive fatty acid tracers facilitated direct measurement of trafficking of dietary fat throughout the body. Daily treadmill exercise not only increased dietary fat oxidation but also decreased the trafficking of dietary fat to RP and SC adipose depots during relapse (Figures 3A,B, respectively). Unlike dietary fat oxidation, this shift in fat trafficking appears to be driven by the energy balance state of the animal as dietary fat retention in the visceral adipose depots was similar in the REL-EX and REL-GM groups in both adipose depots. However, in interpreting this data, it is also necessary to account for the size of each adipose depot. While not statistically different, we did see a trend for a decrease in the mass of both RP and to a lesser extent SC adipose depots in the REL-GM group, compared to the REL-EX (Table 1). Thus, while there are no differences in dietary fat retention at the whole tissue level, we have *previously* shown higher dietary fat retention in the REL-GM compared to the REL-EX on a per gram level (Steig et al., 2011), despite the fact that the REL-EX are eating more to achieve the same positive energy imbalance as the REL-GM during relapse.

**Table 1:** **Characteristics of RP and SC adipose depots**.

	**Obese**	**Reduced**		**Relapsing**		
		**SED**	**EX**	**SED**	**EX**	**GM**
**RP ADIPOSE**						
Mass	21.7±1.6	6.7±1.1	8.3±1.8	11.3±1.3	10.2±1.0	8.4±0.8
Mean adipocyte diameter	119.9±0.8	76.6±0.7	72.7±0.8	83.7±0.8	76.3±0.7	77.6±0.6
Number of adipocytes (×10^6^ cells)	24.0±1.9	26.0±3.4	31.1±1.0	29.1±3.5	34.1±3.2	33.9±3.9
**SC ADIPOSE**						
Mass	210.6±7.6	143.0±8.5	140.7±8.9	148.7±7.8	150.2±9.2	146.4±7.1
Mean adipocyte diameter	84.2±0.8	68.3±0.8	54.8±0.6^*^	68.6±0.7	56.3±0.6^*^	59.7±0.5
Number of adipocytes (×10^9^ cells)	0.57±0.09	0.83±0.23	1.43±0.18^*^	0.76±0.11	1.12±0.09	1.07±0.12

*RP and SC adipose depots were weighed, a portion was collagenase digested, and adipocyte diameter was measured. The number of cells per fat pad was calculated with the average adipocyte volume, a density conversion factor (0.915 g/mL), and the mass of the fat pad. All data represent mean ± SEM*.

* *p < 0.05 SED vs. EX, within either the reduced or relapsing group*.

### *De novo* lipogenesis

Incorporation of tritium into lipid pools was used to estimate the net retention of carbons via lipogenesis, with the majority of this being *de novo* lipogenesis. In the weight reduced state, very little *de novo* lipogenesis is expected as the animals are eucaloric. Thus, it was not surprising that ^3^H incorporation into both RP and SC adipose was low in weight reduced animals, and unaffected by exercise (Figures 3C,D). In relapsing animals, however, exercise significantly decreased ^3^H retention and presumably *de novo* lipogenesis in RP adipose (REL-SED vs. REL-EX; Figure 3C). Surprisingly, ^3^H retention was significantly lower in RP adipose, and tended to be lower in SC adipose (*p* = 0.09) of REL-GM rats compared to the REL-EX group.

### Gene expression data

To further explore the mechanisms by which exercise may contribute to the shunting of dietary fat away from storage and toward oxidative tissues during relapse, we analyzed RP adipose tissue for expression of several genes involved in lipid uptake, synthesis and *de novo* lipogenesis, as well as transcription factors that regulate the expression of these genes.

#### Transcription and regulatory factors

**Figure 4:**
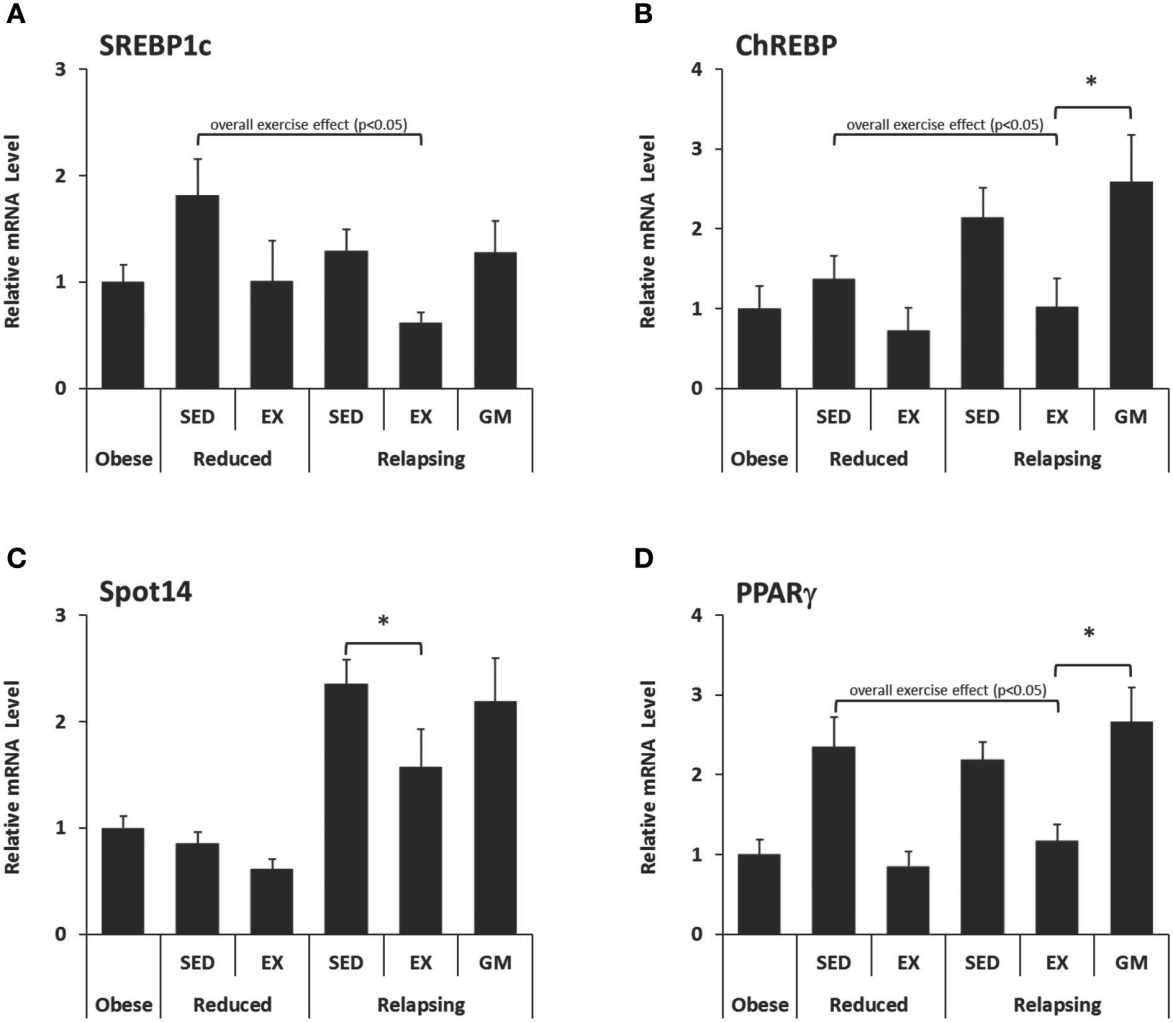
**mRNA Expression of regulators of lipid metabolism in retroperitoneal adipose tissue**. Expression of **(A–C)** Regulators of *de novo* lipid synthesis and **(D)** a transcription factor involved in adipogenesis, lipid uptake, and storage were measured in RP adipose tissue by qPCR and normalized to the expression of ubiquitin C using the comparative Ct method. (^*^P < 0.05).

Expression of three regulators of *de novo* lipid synthesis (Figures 4A–C), and one transcription factor involved in adipogenesis, lipid uptake and storage (Figure 4D) were measured in RP adipose tissue. In both the weight-reduced and relapsing states, exercise decreased the expression of sterol response element binding protein (SREBP-1c; Figure 4A), carbohydrate-responsive-element-binding protein (ChREBP; Figure 4B), and peroxisome proliferator-activated receptor γ (PPARγ; Figure 4D). A reduction in thyroid hormone responsive spot 14 (Spot 14; Figure 4C) with exercise was limited to relapsing animals. This decrease in transcription factor expression was not seen in the REL-GM group, indicating that these effects were a direct result of exercise.

#### Lipid uptake

**Figure 5:**
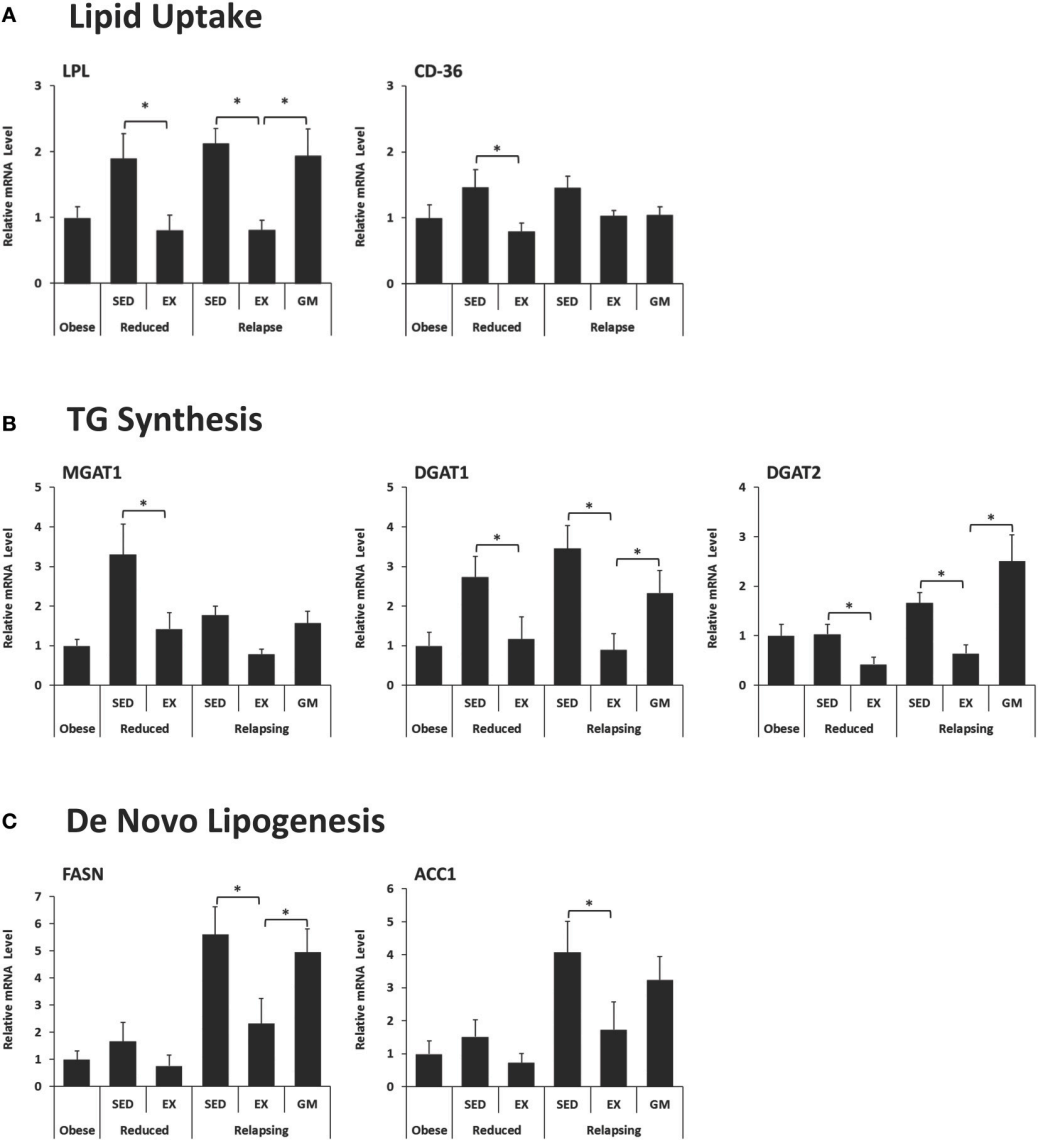
**Expression of genes in retroperitoneal adipose tissue that regulate**. **(A)** Lipid Uptake, **(B)** Triglyceride (TG) synthesis, and **(C)** *De novo* lipogenesis. Expression was measured in RP adipose tissue by qPCR and normalized to the expression of ubiquitin C using the comparative Ct method. (^*^P < 0.05).

Exercise decreased the expression of lipoprotein lipase (LPL) in both weight reduced and relapsing animals (Figure 5A). LPL functions to hydrolyze lipoprotein triglycerides (from chylomicrons and very low density lipoproteins), thereby providing non-esterified fatty acids for tissue use. This decrease in LPL was not observed in the REL-GM group, indicating that regulation of LPL is due to the independent effects of exercise. CD-36, which facilitates fatty acid uptake, was similarly decreased with exercise in both weight-reduced and relapsing rats (Figure 5A). In contrast to LPL, CD-36 was not different between REL-EX and REL-GM rats indicating that changes in CD-36 appear to be mediated by energy balance directly.

#### Capacity for lipid synthesis

Synthesis of triglycerides is regulated by a family of enzymes including monoacyglycerol and diacylglycerol acyltransferases (MGAT and DGATs, respectively). In this model, expression of MGAT1, DGAT1, and DGAT2 decreased with exercise in both WR and REL groups (Figure 5B). While the exercise-induced decrease in MGAT1 only reached significance in weight-reduced animals, the same trend occurred in relapsing animals. DGAT1 and DGAT2 were significantly lower in exercising animals both in the weight reduced and relapsing states. This effect of exercise on the capacity for TG synthesis appears to be due, at least in part to an independent effects of exercise, as the REL-GM rats have higher expression of DGAT1/2 compared to the REL-EX rats (*P* < 0.05 for both).

#### Capacity for *de novo* lipogenesis

Exercise was associated with decreased expression of both fatty acid synthase (FAS) and acetyl-CoA carboxylase 1 (ACC1) (Figure 5C), mirroring the decrease in *de novo*-derived lipid measured in this tissue via ^3^H incorporation (Figure 3C). Interestingly, comparison of the REL-EX and REL-GM groups shows that while expression of FAS is higher in RP adipose from REL-GM compared to REL-EX, our estimates of *de novo* lipogenesis in these two groups show the opposite trend. Given this discrepancy, we examined ^3^H incorporation into other depots, including the SC adipose, skeletal muscle, and liver. As reported previously (Steig et al., 2011), ^3^H incorporation in the liver was significantly higher in the relapsing animals during exercise, compared to their energy gap-matched controls (REL-EX vs. REL-GM: 7.3 ± 0.7 vs. 4.3 ± 0.5 ηCi/g tissue, *P* < 0.05). Our interpretation of this data is that with exercise, a portion of the *de novo*-derived fat measured in the RP fat pad may, in fact, be synthesized in the liver and then transported to the visceral adipose depots for storage. Not only would this process require additional energy, it would also provide a second opportunity for this fat to be oxidized by the skeletal muscle when it is released into circulation as VLDL.

### Integrated responses: muscle and adipose

**Figure 6:**
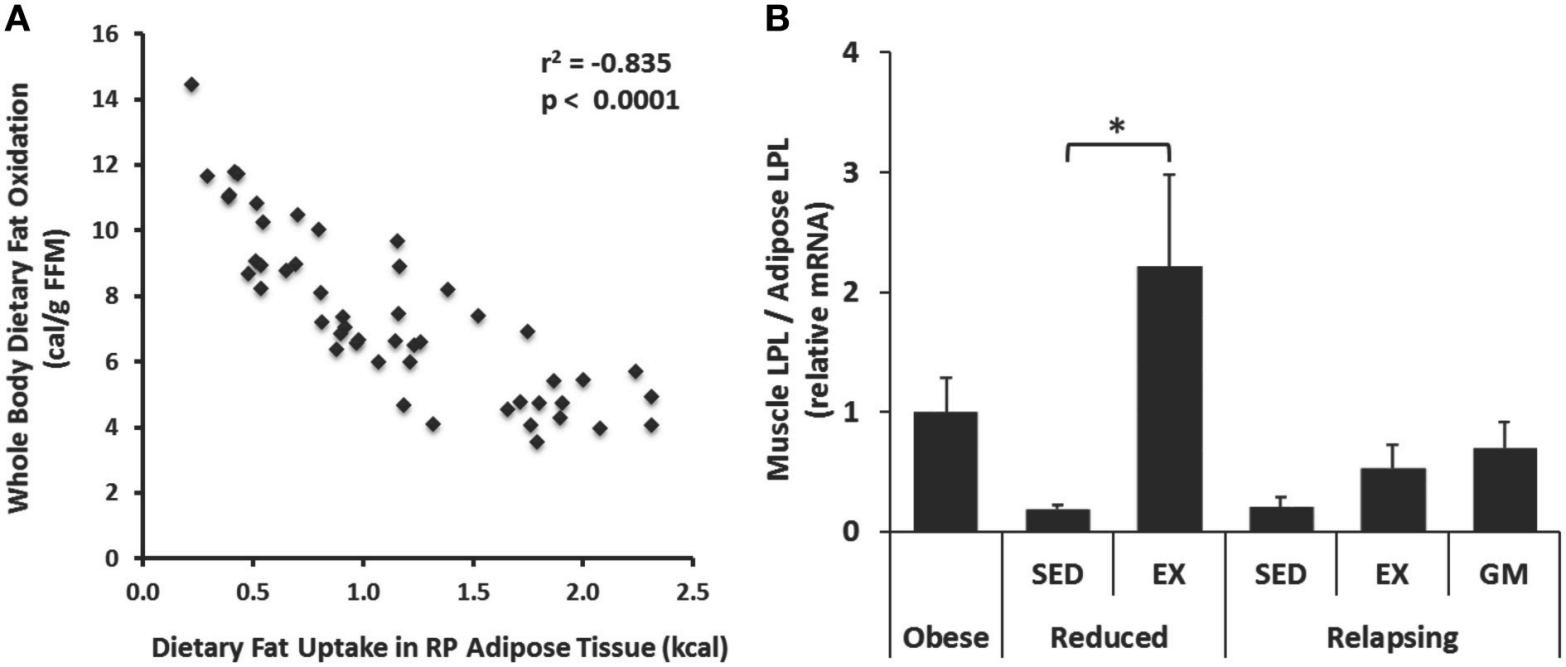
**Relationships between muscle and adipose tissue**. **(A)** Whole body dietary fat oxidation was inversely correlated with trafficking of dietary fat to RP adipose tissue. **(B)** Exercise increased the expression of lipoprotein lipase (LPL) in skeletal muscle (gastrocnemius) relative to RP adipose both in the weight reduced state, and following 1 day of relapse. Expression was measured by qPCR. (^*^P < 0.05).

While our goal in the current study was to identify exercise-induced changes in adipose tissue that could affect metabolism, it is important to point out the interaction between skeletal muscle and adipose in the trafficking of dietary fat and other aspects of whole body metabolism. For example, dietary fat storage in adipose tissue was inversely correlated with whole body dietary fat oxidized (Figure 6A), which may reflect how integrated these two parameters are across metabolic states. Further, in our original publication (Steig et al., 2011), we reported that muscle LPL mRNA was elevated with exercise in the weight reduced state. The differential expression of LPL in muscle and adipose tissue is thought to play a significant role in trafficking of nutrients. Here, we combined these datasets to examine the ratio of LPL mRNA expression in these tissues. Expectedly, this ratio was clearly affected by regular exercise in a manner that would promote the trafficking of dietary fat toward muscle and away from adipose tissues (Figure 6B).

### Adipocyte cellularity

**Figure 7:**
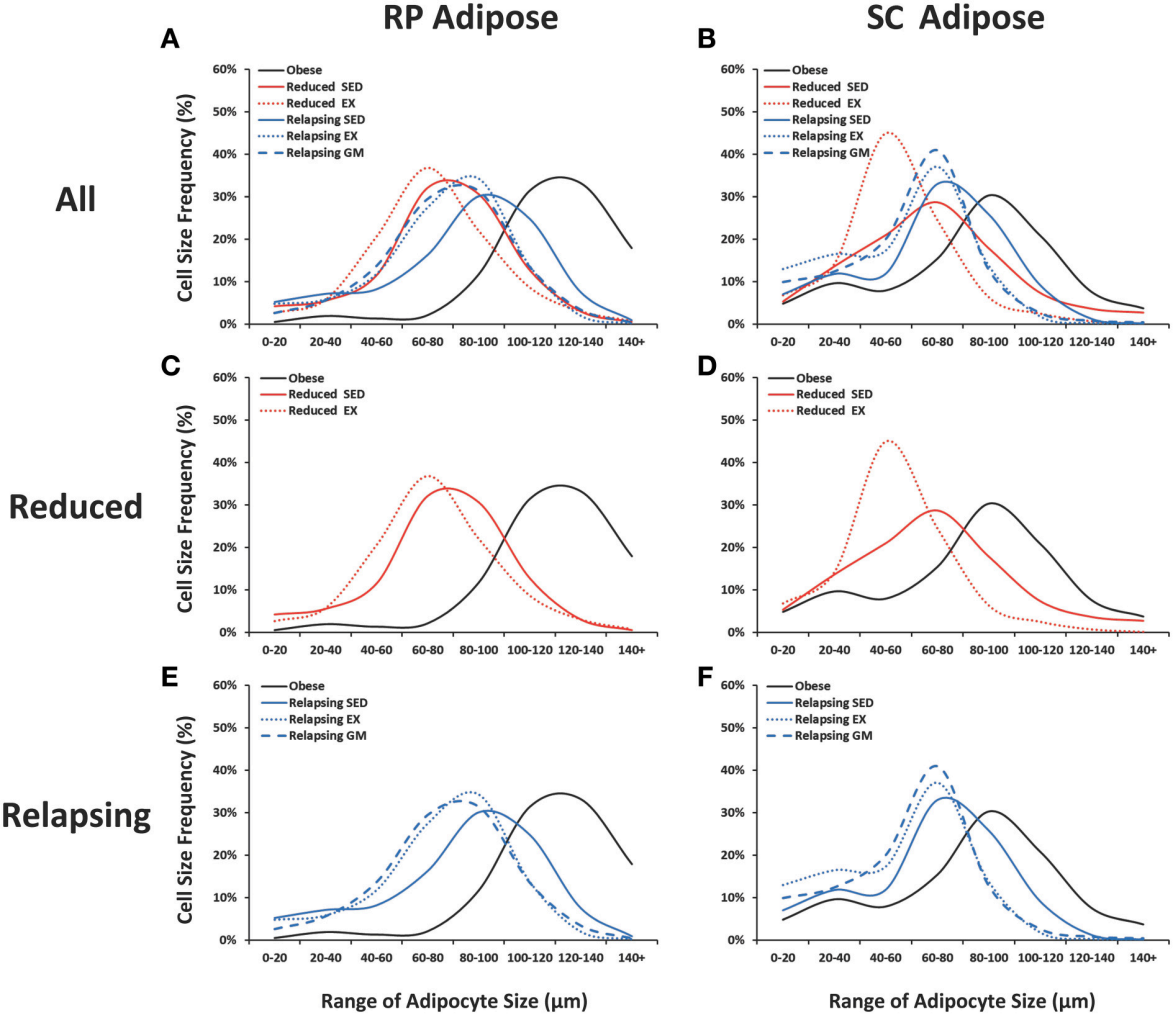
**Adipocyte size frequency distributions**. Retroperitoneal (RP) and subcutaneous (SC) adipose tissue were collagenase digested and adipocyte diameter was measured.Frequency distributions are shown for all groups **(A,B)**, weight reduced animals (**C,D**; red) and relapsing animals (**E,F**; blue). Sedentary animals are represented with a solid line; exercising animals with a dotted line, and gap-matched animals with a dashed line.

As we have previously reported (Maclean et al., 2011), sustained weight loss reduces average adipocyte size and shifts the size frequency distribution of adipocytes. In this study, we examined how regular exercise affected the cellularity characteristics of RP and SC fat pads during weight loss maintenance and during the initial day of weight regain (Figures 7A,B). Exercise had no effect on the mass of the RP and SC depots in the weight reduced or relapsing rats (Table 1), but it did have distinct effects on the cellularity characteristics. In the weight reduced state, exercise exacerbated the left-shift in the frequency distribution profile of both depots, an effect that was particularly striking in the SC depot (Figures 7C,D). This shift toward smaller adipocytes was accompanied by an increase in the total number of adipocytes in both RP and SC depots of weight-reduced, exercised rats (Table 1). This left shift was maintained with exercise during relapse (Figures 7E,F). In the RP depot, profiles of REL-EX and REL-GM were superimposed, suggesting that the smaller adipocyte size in the exercising animals was primarily due to attenuated overfeeding in these groups, when compared to their sedentary counterparts. However, in the SC depot, exercise significantly increased the number of very small adipocytes (<60 μm) during relapse when compared to their gap-matched controls (REL-EX vs. REL-GM), suggesting that this depot may be particularly responsive to the effects of exercise, beyond the energy-balance effects seen in the RP depot.

## Discussion

The novel observation of this study is that regular exercise attenuates the expression of adipose lipogenic genes during weight regain, an effect which coincides with the trafficking of dietary fat toward oxidative pathways, presumably in skeletal muscle. Much of this concerted impact of exercise on regulators and pathway enzymes of lipid metabolism remained significant even when compared to sedentary gap-matched controls. Taken together with our previous findings (Steig et al., 2011), these data suggest that regular exercise affects both muscle and adipose tissues during weight regain to redirect the deposition of excess energy. Related to this preferential nutrient trafficking is a reduction of the energy gap between appetite and expended energy. Our working hypothesis is that the tissue-specific effects of exercise on adipose tissue and muscle alter peripheral metabolism in a way that contributes to the reduced energy gap in the weight reduced state. The fact that exercise counters the biological drive to regain weight and reduces this energy may explain its beneficial effects for weight loss maintenance in humans (Ewbank et al., 1995; Schoeller et al., 1997; Weinsier et al., 2002; Jakicic et al., 2008; Catenacci et al., 2011).

More specifically, our examination of skeletal muscle gene expression in these animals (Steig et al., 2011) revealed exercise-induced increases in expression of genes that favor the uptake, mobilization, and oxidation of fat, which likely contributed the preferential oxidation of ingested fuels upon overfeeding. The current study extends these findings to reveal that adipose tissue is not merely a passive storage site for excess nutrients. Rather, exercise affects gene expression in adipose tissue in a manner that inhibits lipid uptake, triglyceride synthesis, and *de novo* lipogenesis. This does not necessarily mean that adipose tissues are not capable of taking up nutrients during overfeeding. However, it may redirect excess nutrients to other tissues and alternative metabolic pathways prior to being deposited. Together this interplay between muscle and adipose tissue plays an important role in coordinating the trafficking of nutrients and mediating the metabolic effects of exercise after weight loss.

One of the surprising findings of this study was that despite having significantly lower expression of lipogenic genes, the net retention of *de novo*-derived lipid was higher in both the RP and SC adipose depots of exercising animals compared to their energy gap-matched controls. Our interpretation of this data is that much of this lipid is being made by the liver and subsequently trafficked to adipose tissue storage. Using ^3^H data does not allow us to determine where lipid is being made, only where it is stored. However, this perspective of fuel trafficking would be consistent with exercise's promotion of dietary fat oxidation and the ^3^H enrichment of lipid pools in adipose tissue, skeletal muscle, and liver, which we have previously reported (Steig et al., 2011). The trafficking of excess nutrients through *de novo* pathways in the liver would have two beneficial consequences in terms of weight maintenance and regain: (1) it would increase the energetic cost of depositing excess energy, which we observed as TEE was elevated beyond the energetic cost of the exercise bout, and (2) it would provide a second opportunity for these excess nutrients to be oxidized—this time as newly formed very low density lipoproteins that could be taken up by oxidative tissues, like skeletal muscle, while en route to their final storage depot.

Our data also indicate that exercise-induced changes in adipocyte size and number may be an additional mechanism by which exercise could facilitate long term weight reduction and/or improve metabolic health when weight regain occurs. When compared to larger adipocytes (at least *in vitro*) smaller adipocytes are more sensitive to the anti-lipolytic effects of insulin, exhibit lower basal and catecholamine-induced lipolysis, have a lower lipid turnover, and express genes favoring energy storage (Bjorntorp et al., 1975; Lofgren et al., 2005; Svensson et al., 2008). In the present study, we found that exercise increased the number of small, and decreased the number of large adipocytes, compared to sedentary controls. This effect was particularly apparent in SC adipose depots, which may contribute to the exercise-induced shift in nutrient trafficking to peripheral adipose tissues. We know that expanding visceral adipose depots is associated with increased risk for diabetes, cardio-metabolic disease, and all-cause mortality (Milewicz et al., 2001); however subcutaneous adipose tissue, even in obese individuals, appears to have a more benign phenotype, and shows no independent associations with insulin resistance or cardiovascular disease risk (Neeland et al., 2013). Thus, if exercise can promote the storage of nutrients in SC, rather than visceral fat, this would represent a further beneficial effect of exercise—in this case not to prevent weight regain, but rather to improve metabolic health in those that do gain weight.

Despite this potential benefit of exercise on adipose tissue metabolism, one must consider the potential consequences that an increase in adipocyte number could have on the long term expansion of body fat stores. Increased adipocyte hyperplasia may be of little concern if an individual is in a weight reduced state; however, upon cessation of exercise and/or overfeeding, these inulin sensitive cells are primed to take up and store triglyceride. Thus, this may increase the propensity for weight gain if the exercise regimen is stopped. While not well documented in the scientific literature, some (Elleuch et al., 2008; Arliani et al., 2014), but not all (O'Kane et al., 2002), studies have reported higher amounts of weight gain in former collegiate and/or professional athletes later in life. While there are numerous factors that could lead to weight gain in former athletes, exercise-induced changes in adipocyte cellularity is one possibility worth considering.

The changes in adipose tissue gene expression reported here reflect one snap shot in time, at the end of a 24-h study. Weight regain is a dynamic, non-steady-state process in which both gene expression and metabolic flux are likely to vary during the early stages of the relapse to obesity. The importance of this effect on adipose tissue during the weight regain process is likely to depend on the magnitude and persistence of this effect. Furthermore, our studies were focused on male obese rats, and both the biological response to weight loss and the effects of exercise may be different in females. Finally, while our studies show which adaptive responses in adipose tissue gene expression are being attenuated during weight regain, the design of our studies do not reveal an underlying mechanisms of how exercise might impose this effect. These issues and limitations are being addressed in ongoing studies. Further, subsequent studies will be needed to translate these observations to humans, where individual variability in the response to exercise are compounded by compensatory behavioral changes in daily activity and food intake (MacLean et al., 2015b).

A limitation of this follow-up study in relapsing rats is that we do not have a clear understanding of the underlying mechanisms for the benefits of exercise. One possibility is that the exercise leads to an increase in sympathetic tone in adipose tissues, and the differential expression of adrenergic receptors in the depots may have contributed to the depot-specific effects on gene expression and cellularity. Some studies suggest that SNS activation inhibits lipolysis in SC adipose that tends to be enriched with α_2_ receptors, while increasing lipolysis in visceral fat, where there are more β3-receptors (reviewed in McMurray and Hackney, 2005). Alternatively, exercise-induced changes in inflammation could also mediate some of the adaptive responses. Specifically, wheel running has been shown to decrease the number of crown-like structures (a marker of macrophage infiltration) in mice fed an atherogenic diet, and this was associated with an increase in the number of small adipocytes, and an overall improvement in metabolic outcomes and adipose tissue function (Haczeyni et al., 2015). Finally, there is an emerging body of data demonstrating that weight loss and resulting decrease in adipocyte size imparts changes in the extracellular matrix (ECM) that can cause mechanical stress between the ECM and surrounding adipocytes. This mechanical stress is thought to impose adaptive responses in adipocytes that contribute to subsequent weight regain (Mariman and Wang, 2010; Mariman, 2012; Roumans et al., 2015). At present, we do not know if the impact of exercise on adipocyte cellularity counters this mechanical stress and its metabolic consequences. It is clear that we need further studies to understand the molecular mechanisms underlying the beneficial effects of exercise on adipose tissues in the weight reduced state.

In summary, weight loss from caloric restriction induces an integrated response in a number of tissues in the body that coordinately lead to a strong drive to overeat, metabolize ingested nutrients in an energetically efficient manner, and regain lost weight. Adipose tissues not only contribute to this biological response to weight loss, but also appear to be mediating some of the beneficial countermeasures of exercise during weight regain. These specific effects of exercise in adipose tissue explain why regular exercise is so critical for successful weight loss maintenance.

## Author contributions

EG participated in the design and execution of the study, conducted data analysis and interpretation, and drafted the manuscript; AS participated in the design and execution of the study; MJ participated in the design and execution of the study and assisted with data analysis and interpretation; JH participated in design and execution of the study and interpretation of the data; GJ participated in execution of the animal study; RL assisted with data collection; PM contributed to the design, execution, and data analysis/interpretation. All authors read and approved the final manuscript.

## Funding

This work was supported by the National Institutes of Health (DK038088 and HD073063 to PM). EG was supported by K99 CA169430 and a pilot award from the Colorado Nutrition Obesity Research Center (DK48520 to James O. Hill). We appreciate the generous support from the Energy Balance and Metabolic Core Laboratories within the Colorado Nutrition Obesity Research Center. Additional research support was provided by the Colorado Obesity Research Institute.

### Conflict of interest statement

The authors declare that the research was conducted in the absence of any commercial or financial relationships that could be construed as a potential conflict of interest.
